# Cell Type-Specific Properties of Subicular GABAergic Currents Shape Hippocampal Output Firing Mode

**DOI:** 10.1371/journal.pone.0050241

**Published:** 2012-12-10

**Authors:** Gabriella Panuccio, Stefano Vicini, Massimo Avoli

**Affiliations:** 1 Montreal Neurological Institute and Department of Neurology and Neurosurgery, McGill University, Montreal, Quebec, Canada; 2 Department of Pharmacology and Physiology, Georgetown University, Washington, D.C., United States of America; Georgia State University, United States of America

## Abstract

GABAergic function of the subiculum is central to the regulation of hippocampal output activity. Subicular neuronal networks are indeed under potent control by local inhibition. However, information about the properties of GABAergic currents generated by neurons of this parahippocampal area in normal tissue is still missing. Here, we describe GABA_A_ receptor (GABA_A_R)-mediated phasic and tonic currents generated by principal cells (PCs) and interneurons (INs) of the rat subiculum. We show that in spite of similar synaptic current densities, INs generate spontaneous IPSCs (sIPSCs) that occur less frequently and exhibit smaller charge transfer, thus receiving less synaptic total current than PCs. Further distinction of PCs between intrinsically bursting (IB) and regular-spiking (RS) neurons suggested that sIPSCs generated by the two PC sub-types are likely to be similar. PCs and INs are also controlled by a similar tonic inhibition. However, whereas a comparable tonic current density is found in RS cells and INs, IB neurons are constrained by a greater inhibitory tone. Finally, pharmacological blockade of GABA_A_R did not promote functional switch of RS neurons to IB mode, but influenced the bursting propensity of IB cells and released fast spiking activity in INs. Our findings reveal differences in GABAergic currents between PCs and INs as well as within PC sub-types. We propose that GABAergic inhibition may shape hippocampal output activity by providing cell type-specific fine-tuning of subicular excitatory and inhibitory drives.

## Introduction

The subiculum represents the major hippocampal output. It funnels information flow from the hippocampus proper to para- and extra-hippocampal areas, thus influencing cognitive and physiological functions, such as learning and memory, stress responses and the generation of rhythmic brain activity [1], [2]. The role of the subiculum in gating hippocampal output is largely contributed by its intrinsic GABAergic function: local GABAergic signalling restrains the propagation of afferent excitation [3] and modulates the firing behavior of subicular principal cells (PCs). In particular, a strong control by local inhibition is seen in intrinsically bursting (IB) neurons that may provide a major contribution to subicular output activity [4]. The subiculum is indeed regarded as a bursting structure. However, the functional versatility of this parahippocampal area also resides in the presence of a wide variety of electrophysiologically distinct cell types [5]. Besides IB and regular spiking (RS) cells, which are projection neurons, the subiculum is populated by a variety of interneurons (INs), most of which exhibit fast-spiking activity [6]. As in other brain regions, the many electrophysiological classes of subicular cells may exert distinct functional roles. IBs, RSs and INs are reciprocally connected to constitute elementary neuronal ensembles in which these three cell classes play a defined role in the generation and maintenance of local and distant population activity [6].

Differences in inhibition of distinct cell classes are seen in the hippocampus [7], as well as in the amygdala [8], the thalamus [9], [10], the cerebellum [11] and the neocortex [12]. Moreover, a wealth of evidence has recently emerged supporting the relevance of impaired subicular GABAergic signalling with regard to several neurological conditions, including mental retardation [13] and epilepsy [14]–[17]. However, information on the properties of GABA_A_R-mediated currents generated by subicular cells in the normal brain is still missing. In light of this evidence, we hypothesized that GABA_A_R-mediated currents may be differentially expressed in subicular cells, and we further argued that cell type-specific properties of GABAergic currents modulate subicular output activity. We therefore performed somatic whole-cell patch-clamp recordings from rat brain slices to characterize both phasic and tonic inhibition of subicular cells. We report that differences in GABAergic currents not only exist between PCs and INs, but also within PC sub-types.

## Methods

### Ethics Statement

All procedures were conducted in compliance with the guidelines provided by the Canadian Council on Animal Care and approved by the Animal Care Committees of McGill University and the Montreal Neurological Institute (Animal Use Protocol 1562). All efforts were made to minimize the number of animals used and their suffering.

### Brain slice preparation and maintenance

Combined horizontal brain slices (300 µm thick) comprising the hippocampal formation were obtained from 37 male, Sprague-Dawley rats (Charles River, St-Constant, Qc, Canada) aged 2–3 months, 250–350 g. Animals were sedated with isoflurane, then deeply anesthetized with a Ketamine/Xylazine cocktail (90/10 mg/Kg, i.p.) and transcardially perfused with ice-cold (2–4°C) sucrose-based artificial cerebro-spinal fluid (sucrose-ACSF, *cf.*, [18]) composed of (mM): Sucrose 206, KCl 3.5, MgSO_4_ 2, NaH_2_PO_4_ 1.25, MgCl_2_ 1, CaCl_2_ 1, NaHCO_3_ 26, D-Glucose 10, L-Ascorbic Acid 1, Kynurenic Acid 1, Pyruvic Acid 3. Animals were then decapitated, their brains quickly removed and let chill for ∼3 min in ice-cold carbogenated sucrose-ACSF. Horizontal brain slices were cut with a Leica VT1000S vibratome (Leica, Nussloch, Germany), immediately placed in a custom-made submerged holding chamber and let recover at room temperature (∼21°C) for ≥1 hr in ACSF composed of (mM): NaCl 124, KCl 3.5, MgSO_4_ 2, NaH_2_PO_4_ 1.25, CaCl_2_ 2, NaHCO_3_ 26, D-Glucose 10, L-Ascorbic Acid 1 and supplemented with Pyruvic Acid, 3 mM. All extracellular solutions were equilibrated at pH ∼7.35 with O_2_/CO_2_ 95/5% gas mixture and their osmolarity was 295–305 mOsm.

### Whole-cell patch-clamp recording

Individual slices were placed into a submerged recording chamber (RC-27L, Warner Instruments LLC, Hamden, CT) mounted on a fixed stage and continuously perfused at ∼1.5 ml/min with ACSF. Pharmacologically isolated GABA_A_R-mediated currents were recorded at room temperature (∼21°C) in the presence of the ionotropic glutamatergic antagonists CNQX (10 µM) and CPP (10 µM), and the GABA_B_R antagonist CGP 55845 (4 µM).

Subicular neurons were visualized with video-enhanced IR-DIC and patched at a depth of >50 µm to minimize decrease in synaptic input that may occur in superficial neurons [19]. Since our study did not aim at corroborating the bursting nature of the subiculum, no systematic approach was used in selecting neurons within this structure; rather, cells were randomly patched from its pyramidal cell layer.

Patch pipettes (tip diameter 2.0–3.0 µm, tip resistance 2.7–3.3 MΩ) were pulled from thick-walled borosilicate glass capillaries (1.5 mm o.d., 0.86 mm i.d., Harvard Apparatus, Holliston, MA, USA) using a Sutter P-97 puller (Sutter Instruments, Novato, CA). Pipettes were filled with the following solution (mM): KCl 120, K-Gluconate 5, EGTA 10, HEPES 10, MgCl_2_ 2, CaCl_2_ 1, ATP-Na_2_ 2, GTP-Na_3_ 0.4, pH 7.20 with KOH 1 M, 280–290 mOsm.

Whole-cell patch-clamp recordings were performed in current- or voltage-clamp mode with a Multiclamp 700A amplifier connected to the Digidata 1322A (Molecular Devices, Sunnyvale, CA, USA). Recordings were performed at a holding potential V_h_ = −70 mV and were started ≥10 min after membrane patch rupture to allow complete cell dialysis. Series resistance (R_s_) was monitored throughout the experiment and recordings were discarded if R_s_>20 MΩ or if it increased by ≥25%. Cells with V_m_ more depolarized than −50 mV were discarded *a priori*. All drugs were bath-applied and delivered through a VC-6 pinch-valve perfusion system (Warner Instruments LLC, Hamden, CT).

### Chemicals and drugs

All chemicals and drugs were purchased from Sigma-Aldrich Canada (Oakville, ON, Canada) except for CGP 55845, CNQX and CPP (Tocris Bioscience, Ellisville, MO, USA).

### Data and statistical analyses

Traces were acquired with the software pClamp 8.2 (Molecular Devices, Sunnyvale, CA, USA) and stored on the hard drive for off-line analysis. Current-clamp recordings were sampled at 20 kHz, whereas voltage-clamp recordings were sampled at 10 kHz and low-pass filtered at 1–2 kHz off-line.

The apparent input resistance (R_in_) was measured according to Ohm's law by means of hyperpolarizing current-step protocols (first step: −0.2 nA, 0.02–0.04 nA increment, 500 ms) from current-clamp traces free from contaminant spontaneous inhibitory post-synaptic potentials (sIPSPs). The same traces were used to measure the membrane time constant (τ_m_) by fitting the hyperpolarizing cell responses with a monoexponential function. Membrane capacitance (C_m_) was calculated using the seal test.

The effect of GABA_A_R blockade on burst-firing of IB neurons was assessed by comparing the burst ratio of IB cells responses induced by depolarizing current injection before and after application of picrotoxin (100 µM). A burst was defined as a series of 2 or more action potentials generated at an interval <50 ms and riding on a depolarizing membrane potential fluctuation. We defined burst ratio the ratio between the number of action potentials generated within bursts and the total number of action potentials generated in response to injection of direct positive current.

Spontaneous inhibitory post-synaptic currents (sIPSCs) were analyzed with Mini Analysis 6.0 Software (Synaptosoft, Decatur, GA, USA). Threshold for automatic sIPSC detection was set at 5 times the RMS noise and detected events were accepted or rejected by visual inspection. The average IPSC was then used to measure half-width, charge transfer (Q), rise time constant (τ_R_) and weighted decay time constant (τ_DW_). The latter was calculated as Q/amplitude (*cf.* [9]). The average total current (*I*_tot_) was defined as Q * frequency (Hz) (*cf.* [9]).

The tonic inhibitory current was measured with the use of Clampfit 9.2 software (Molecular Devices) as the difference in the holding current before and after drug application by means of gaussian fit of all-point histograms performed on 300 ms periods free from contaminant sIPSCs. Stationary noise analysis of the tonic current was performed as reported in [19].

Data sets were first tested for normal distribution (Shapiro-Wilk test) and for homoscedasticity (Levene's test), then compared with either of the Student's t-test for paired data or one-way ANOVA followed by Fisher's LSD (protected) or Games-Howell post-hoc test, as appropriate. Data were considered significantly different if p<0.05. Throughout the text, data are expressed as mean ± SEM, and *n* indicates the number of patched neurons, unless otherwise stated.

## Results

### Electrophysiological identification of subicular cell types

**Figure 1:**
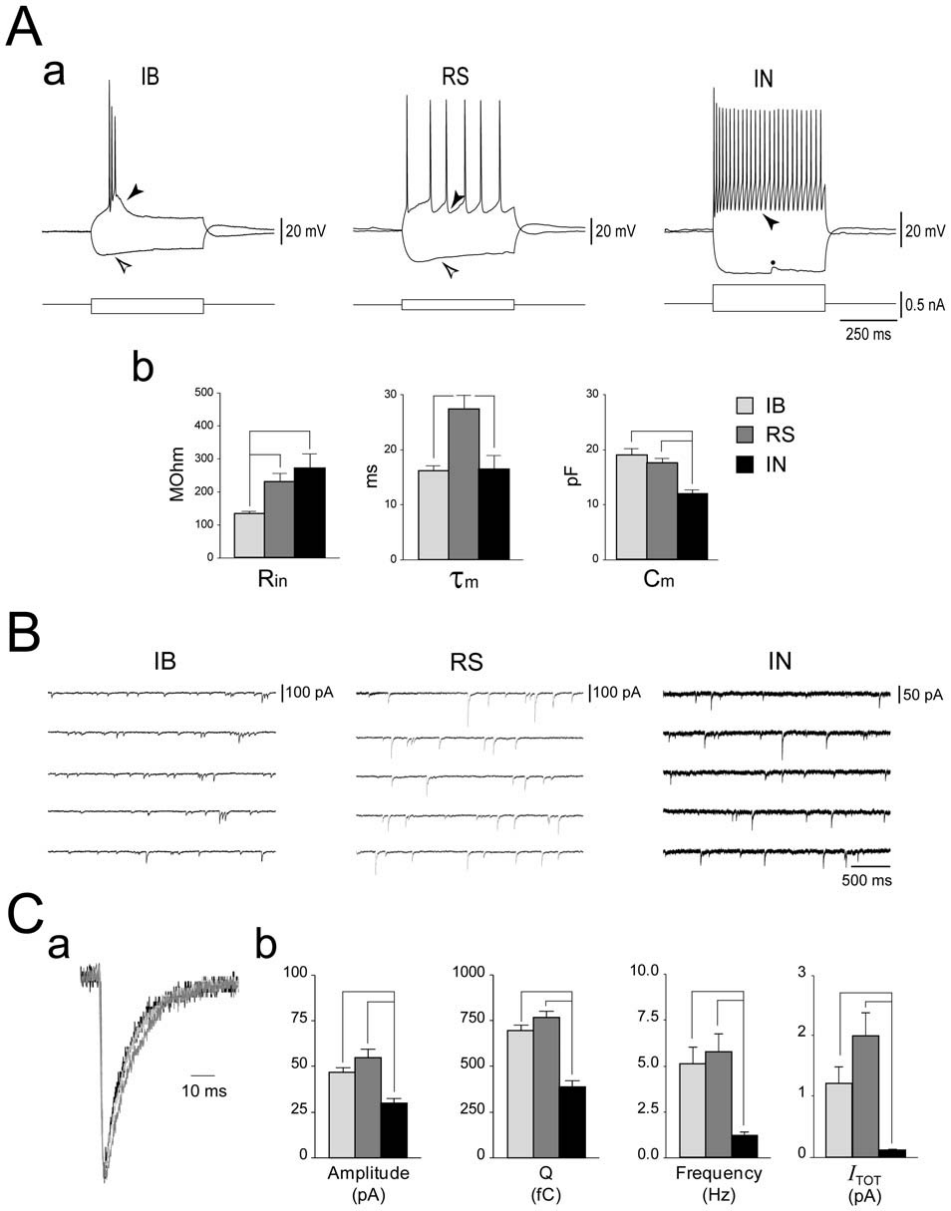
Synaptic inhibition in electrophysiologically identified subicular cell classes. **Aa:** Electrophysiological identification of subicular cells. Whole-cell current-clamp recordings showing the responses of subicular cells to depolarizing and hyperpolarizing current steps. Pulse width: 500 ms, Vm = −70 mV. Two classes of principal cells can be distinguished. When depolarized, the intrinsically bursting neuron (IB) generates a burst of 3 action potentials riding on a depolarizing envelope and followed by a depolarizing after-potential (black arrowhead), whereas the regular spiking (RS) neuron responds with a series of single action potentials followed by an after-hyperpolarization (AHP, black arrowhead). In these sample recordings, both cell types exhibit a *sag* (white arrowhead) when hyperpolarized. The fast-spiking interneuron (IN) responds to depolarizing current injection with high-frequency tonic firing of action potentials, followed by a fast and pronounced AHP (black arrowhead). Note the absence of hyperpolarization-induced sag. The dot indicates an IPSP. **Ab:** Plots summarizing the intrinsic properties of PCs and INs. **B:** *Synaptic inhibition exerts a weaker control on subicular interneurons than on principal cells*. Somatic whole-cell voltage-clamp recordings of sIPSCs generated by principal cells (PC) and interneurons (IN); note the smaller amplitude and the slower rate of occurrence of currents recorded from the IN, as emphasized by the calibration bars. V_h_ = −70 mV. **C:** In (**a**) are the superimposed scaled average sIPSCs generated by the three cell types, in (**b**) the plots emphasize the greater synaptic current received by PCs as compared to INs. Legend in Ab also applies to Cb.

Somatic GABAergic currents were recorded from 70 subicular neurons of the pyramidal cell layer. Detailed analysis was performed for 45 cells that allowed reliable recordings through the various experimental steps. Current-clamp recordings were first performed to identify cell types according to their responses to intracellular current injection (Fig. 1Aa). Thirty-five of these 45 cells exhibited a firing behaviour that was characteristic of subicular principal cells (PCs), whereas the remaining 10 cells were identified as interneurons INs according to [20]. Within PCs, 18 were classified as weak intrinsically bursting (IB) and 17 as regular spiking (RS) neurons (*cf.* [5]).

IB cells did not always fire spontaneous bursts of action potentials, but these could be triggered by intracellular injection of depolarizing current, and were usually followed by a pronounced depolarizing after-potential (DAP, Fig. 1Aa, black arrowhead). However, two IB neurons exhibiting spontaneous bursting responded to depolarizing current injection with RS behavior. Moreover, IBs typically exhibited a prominent *sag* in response to hyperpolarizing current injection (Fig. 1Aa, white arrowhead). RS neurons generated single action potentials that were most frequently followed by a slow after-hyperpolarization (AHP, black arrowhead), whereas a hyperpolarization-induced *sag* (white arrowhead) was not consistently observed. INs were identified by their characteristic generation of short (≤0.5 ms half-width) action potentials followed by a fast, pronounced AHP (Fig. 1Aa, black arrowhead). Six out of 10 INs responded to depolarizing current injection with tonic high frequency firing (>50 Hz), 2 INs generated a single spike followed by subthreshold membrane potential oscillations, and 2 INs exhibited a stuttering firing pattern consisting of irregular generation of action potentials interspersed with subthreshold membrane potential oscillations (see Fig. 3). Moreover, contrary to PCs, a hyperpolarization-induced *sag* was virtually absent in all patched INs. Since these cells may represent similar functional entities (*cf.* [20]), data obtained from them were pooled.

 As summarized in Fig. 1Ab, the apparent input resistance (R_in_) of IB cells (130.2±7.53 MΩ, n = 17) was significantly lower than that of RS neurons (227.04±26.85 MΩ, n = 17, p = 0.006) and INs (268.47±138.54 MΩ, n = 10, p = 0.02). Membrane time constant (τ_m_) of RS neurons (27.23±2.56 ms) was significantly slower than what measured in IB cells (16.07±0.85 ms, p = 0.002) and INs (16.35±2.52 ms, p = 0.01). Moreover, consistent with a smaller cell surface, INs exhibited a smaller membrane capacitance (C_m_) compared to PCs (IN: 11.77±0.74 pF; IB: 18.93±1.1 pF, p<0.001; RS: 17.38±0.85 pF, p<0.001). At variance, V_m_ was similar among the three cell types (IB: −60.13±1.34 mV; RS: −57.36±1.2 mV; IN: −58.14±1.25 mV).

### Synaptic inhibition of subicular principal cells and interneurons

Fig. 1B shows somatic voltage-clamp recordings of sIPSCs generated by an IB, an RS and an IN whereas the superimposed scaled average sIPSCs are shown in Fig. 1Ca. Synaptic events recorded from PCs were larger in amplitude and exhibited a greater charge transfer (Q) than those recorded from INs (Fig. 1Cb), but.current density and Q/C_m_ were comparable among the three cell types (*cf.* Table 1). In addition, sIPSCs generated in PCs occurred more frequently than in INs. Therefore, in spite of similar current densities, PCs were controlled by a greater synaptic inhibition than INs, as revealed by comparison of the average total current (*I*_TOT_) delivered to these cells (Fig. 1Cb, *cf.* Table 1).

**Table 1:** Properties of GABA_A_R-mediated sIPSCs generated by subicular PCs and INs.

	INn = 10 (1590)	PCn = 28 (8672)	IBn = 12 (2416)	RSn = 16 (6256)
C_m_ (pF)	11.77±0.74	17.84±0.75**	18.52±1.33	17.33±0.91
Amplitude (pA)	30.09±2.66	51.47±3.033**	46.84±2.81**	54.93±4.85**
Current density (pA/pF)	2.72±0.39	2.99±0.19	2.66±0.25	3.23±0.28
Q (fC)	389.87±33.04	737.75±24.13**	694.707±32.84**	770.027±33.66**
Q/C_m_ (fC/pF)	35.01±4.67	43.13±2.18	39.31±3.25	45.99±2.89
Frequency (Hz)	1.149±0.23	5.431±0.69**	5.060±0.93	5.709±0.98
Total current (pA)	0.11±0.02	1.66±0.26**	1.21±0.27	1.99±0.38
Half-width (ms)	7.69±0.71	8.92±0.35	8.97±0.34	8.89±0.58
τ_R_ (ms)	1.16±0.07	1.19±0.08	1.21±0.09	1.18±0.12
τ_WD_ (ms)	13.3±1.03	14.69±0.53	15.05±0.57	15.24±1.21

*n* is the number of cells; in parentheses is the number of sIPSCs.

*p<0.05.

** *p≤0.001 vs* INs.

As summarized in Table 1, sIPSCs recorded from the two PC sub-types were generated at similar frequencies and overall exhibited comparable kinetic properties. Nonetheless, it is worth mentioning that these events were characterized by variable τ_DW_, consistent with the expression of different synaptic GABA_A_R assemblies. In particular, we could observe a trend toward faster τ_DW_ in IB cells (range: 12.49–18.86 ms) and INs (range: 8.22–18.96 ms ms) compared to RS cells (range: 10.87–27.8 ms).

### Tonic inhibition of subicular principal cells and interneurons

**Figure 2:**
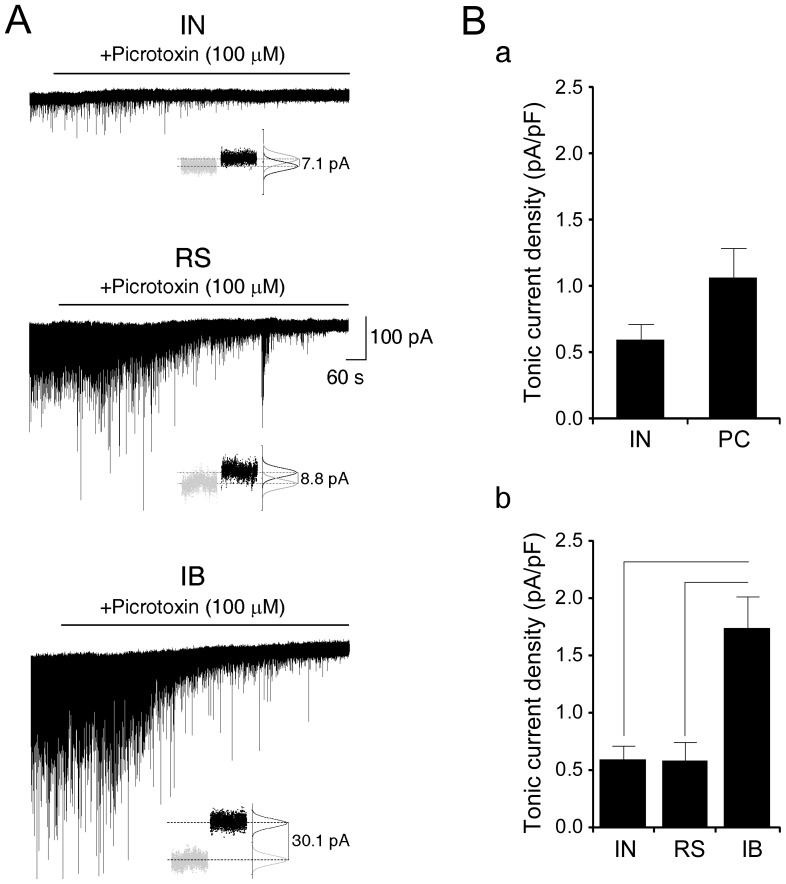
Subicular intrinsically bursting neurons are under greater tonic inhibitory control than regular spiking cells and interneurons. **A:** Somatic voltage-clamp recordings from the indicated subicular cell types (V_h_ = −70 mV). Application of the GABA_A_R blocker picrotoxin (100 µM) abolishes sIPSCs and causes an outward shift of the holding current revealing the expression of a tonic conductance. Insets: the tonic current was calculated by subtracting the mean current values (marked by the dashed lines) obtained during control condition (grey dots) and after application of picrotoxin (black dots) as returned by the normalized gaussian fit of the all-point histograms. **B:** The overall inhibitory tone is similar between PC and IN (**a**). However, as indicated by the plot in (**b**), among IN, RS and IB, the latter generate a greater tonic current.

Application of the GABA_A_R blocker picrotoxin (100 µM) during voltage-clamp recording abolished sIPSCs and caused an outward shift of the holding current, indicating the presence of a tonic GABA_A_R-mediated conductance (Fig. 2A). The tonic current density appeared to be comparable between PCs (1.07±0.7 pA/pF, n = 12) and INs (IN: 0.6±0.12 pA/pF, n = 9) (Fig. 2Ba). However, as summarized in Fig. 2Bb, we found that IBs exhibited a tonic current density (1.75±0.27 pA/pF, n = 5) that was significantly greater than what generated in INs (0.6±0.12 pA/pF, n = 9; p = 0.001) and RS cells (0.58±0.16 pA/pF, n = 7; p = 0.003).

Analysis of current variance (σ^2^), which may be indicative of changes in the biophysical state of the GABA_A_R channel [19], did not evidence a significant difference in picrotoxin-induced changes among the three cell classes (Δσ^2^, IB: 2.16±0.91 pA^2^; RS: 1.18±0.51 pA^2^; IN: 0.61±0.14 pA^2^). However, within each neuronal sub-type, picrotoxin induced a small but significant decrease of current σ^2^ in INs (CTRL: 6.37±0.79 pA^2^, +picrotoxin: 5.76±0.61 pA^2^, p<0.001) and in RS cells (CTRL: 6.53±0.71 pA^2^, +picrotoxin: 5.35±0.54 pA^2^, p = 0.04), whereas changes in current σ^2^ were uneven and overall non significant in IBs (CTRL: 9.69±1.97 pA^2^, +picrotoxin: 7.53±1.21 pA^2^).

Therefore, a cell type-specific expression is also seen in tonic inhibition of subicular cells. Interestingly, as opposed to synaptic inhibition, a smaller inhibitory tone impinges on RS neurons, whereas IB cells are paradoxically under the greatest tonic control.

### Contribution of GABA_A_R-mediated signalling to the firing modality of subicular cells

**Figure 3:**
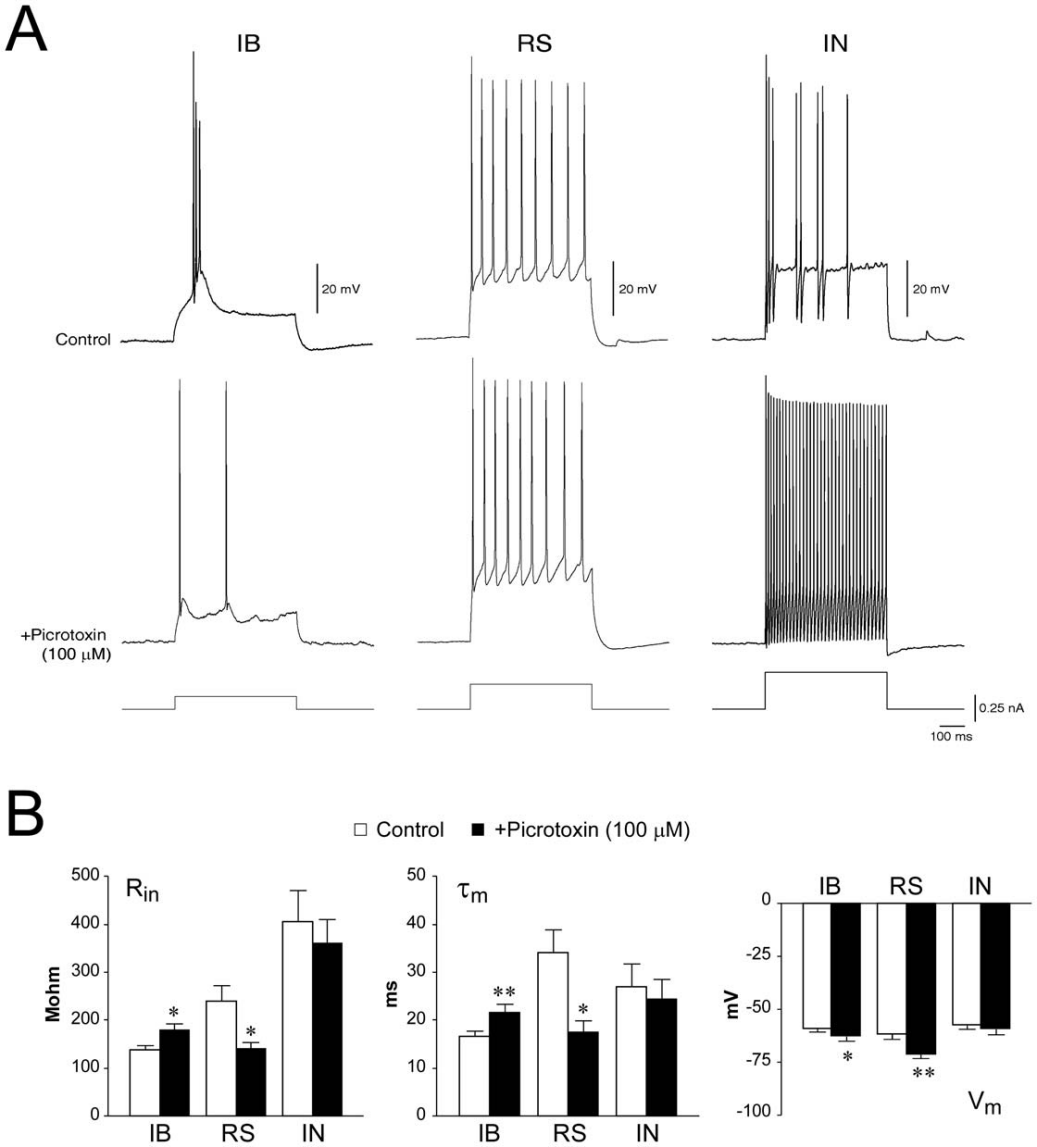
Effect of GABA_A_R blockade on the firing pattern of subicular cells. **A:** Current-clamp recordings showing the effect of GABA_A_R blockade by picrotoxin (100 µM) on the firing modality of an IB, an RS and an IN. Cells were held at V_h_ = −70 mV and depolarized by intracellular injection of 500 ms current pulses (range 0.02–0.26 nA,). Note that, within PCs, only the IB cell changed its firing modality, whereas the same experimental protocol released steady fast-spiking activity of the IN. **B:** Effect of picrotoxin (100 µM) on the intrinsic properties of PCs and INs.

The differences in GABA_A_R-mediated currents among IBs, RSs and INs may modulate the firing modality of these three subicular cell classes (*cf.*, [4], [9]). In order to test this hypothesis, we performed current-clamp recordings to compare the responses of subicular cells to intracellular depolarizing current injection (0.02–0.26 nA, 500 ms) during perfusion with control medium and in the presence of picrotoxin (100 µM) (Fig. 3).

**Figure 4:**
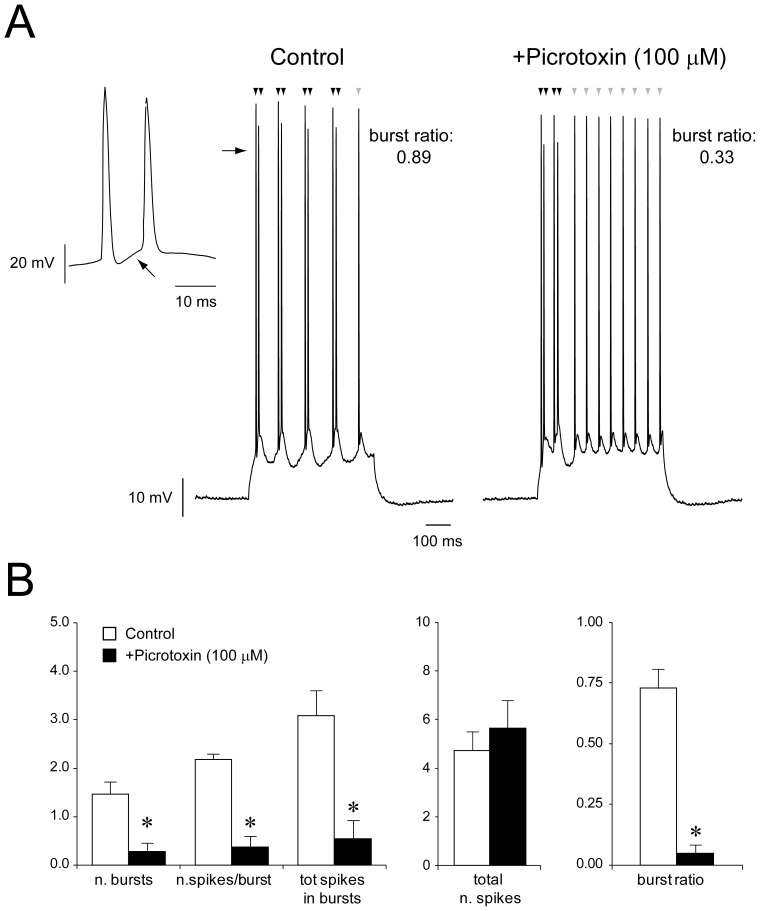
GABA_A_R modulates the bursting propensity of subicular IB cells. **A:** Definition of burst ratio of IB cells responses to depolarising current injection. This IB neuron generated 4 bursts made of 2 spikes (black arrowheads), followed by the generation of a single action potential (grey arrowhead). Therefore, the number of spikes generated during burst-firing was 8 on a total of 9, thus yielding a burst ratio of 0.89. The burst indicated by the horizontal arrow is shown at expanded time scale in the inset on the left, where the black arrow points at the depolarizing membrane potential fluctuation giving rise to the second spike generated within the burst. Bath-application of picrotoxin (100 µM) decreased the burst ratio of this IB neuron to 0.33. **B:** Summary of the parameters used to quantify the bursting behavior of PCs and their changes by pharmacological blockade of GABA_A_R.

Among IB neurons (n = 13), 9 cells could no longer burst and switched to a regular firing pattern when exposed to picrotoxin (Fig. 3A), whereas 2 cells exhibited decreased bursting probability (Fig. 4A). Therefore, this pharmacological procedure significantly decreased the burst ratio of 11/13 IB cells as compared to control condition (CTRL: 0.73±0.08, +PTX: 0.05±0.03, p<0.001, Fig. 4B, *cf.* Table 2). It is worth noting that blockade of GABA_A_R did not affect the total number of spikes generated by IB neurons in response to depolarizing current steps (CTRL: 4.73±0.76, +picrotoxin: 5.64±1.14, Fig. 4B, *cf.* Table 2), thus suggesting that GABAergic signalling influences the bursting propensity rather than the intrinsic firing capability of IB cells (but see also [4]).

**Table 2:** Summary of the effect of picrotoxin (100 µM) on the parameters used to quantify the evoked bursting responses of IB neurons.

	CTRL	+Picrotoxin (100 µM)
**n. bursts**	1.45±0.27	0.27±0.19**
**n.spikes/burst**	2.18±0.12	0.36±0.23**
**tot spikes in bursts**	3.09±0.52	0.54±0.37**
**total n. spikes**	4.73±0.76	5.64±1.14
**burst ratio**	0.73±0.08	0.05±0.03**

** *p<0.001*.

The remaining 2 IB neurons exhibited a variable change of their firing behavior when deprived of the GABAergic drive, therefore making it difficult to quantify these phenomena. Finally, blockade of GABA_A_R significantly increased R_in_ (CTRL: 137.88±10.06 MΩ, +picrotoxin: 178.76±15.15 MΩ; p = 0.02) and τ_m_ (CTRL: 16.63±1.23 ms, +picrotoxin: 21.54±1.81 ms; p = 0.003) in 9 IB cells, whereas V_m_ was overall slightly hyperpolarized (CTRL: −58.93±1.76 mV, +picrotoxin: −62.72±2.18 mV, n = 12, p<0.05).

At variance, the steady-state response pattern of RS cells (n = 5) induced by steady depolarizing current injection was not significantly influenced by GABA_A_R blockade (Fig. 3A), as revealed by analysis of the inter-spike intervals (ISI, CTRL: 97.94±22.05 ms, +picrotoxin: 109.66±0.48 ms). However, picrotoxin significantly hyperpolarized V_m_ of RS cells (CTRL: −61.9±2.1 mV, +picrotoxin: −71.29±1.63 mV, p = 0.002), while significantly decreasing both R_in_ (CTR: 240.5±31.81 MΩ, +picrotoxin: 140.5±14.71 MΩ; p = 0.02) and τ_m_ (CTR: 34.09±4.73 ms, +picrotoxin: 17.4±2.54; p = 0.02; Fig. 3B). The hyperpolarizing effect of picrotoxin seen in PCs is expected, since in our experimental condition (high [Cl^−^]_i_ and V_m_ = −70 mV) GABA_A_R-mediated currents are depolarizing.

Among INs (Fig. 3A), 7/9 cells significantly increased their firing frequency following exposure to picrotoxin (ISI CTRL: 40.06±5.47 ms, +picrotoxin: 27.46±2.96 ms, p = 0.02). The remaining 2 INs did not seem to be affected by this pharmacological manipulation, since they generated a single action potential followed by membrane potential oscillations during both experimental conditions (not shown). As summarized in Fig. 3B, none of the passive membrane properties was affected by picrotoxin treatment (V_m_ CTRL: −57.56±1.85 mV, +picrotoxin: −59.2±2.59 mV; R_in_ CTR: 404.48±66.09 MΩ, +picrotoxin: 360.96±49.79 MΩ; τ_m_ CTR: 26.91±4.96 ms, +picrotoxin: 24.32±0.38 ms).

## Discussion

We performed whole-cell patch-clamp recordings to study the properties of GABA_A_R-mediated currents generated by PCs and INs of the rat subiculum, and to address their contribution to the firing modality of these subicular cell types. We report that both phasic and tonic GABAergic currents exhibit cell type-specific characteristics that likely contribute to modulating the output firing modality of the subiculum.

### sIPSCs differ between PCs and INs

We have shown here that generation of sIPSCs differs among electrophysiologically distinct subicular cell classes. In particular, a weaker synaptic inhibition impinges on INs, as they receive sIPSCs at lower frequency. We have also found that the charge transferred to subicular IB and RS cells is comparable, consistent with experimental evidence indicating that these cells receive similar synaptic inputs [4]. However, we cannot exclude that different classes of INs target the two PC sub-types. In this setting, the distinct intrinsic membrane properties of the two PC sub-types would eventually determine how these inputs are processed (*cf.* [4]), therefore influencing the subicular output pattern.

### A weaker tonic inhibition impinges on subicular INs and differentially modulates the excitatory drive

We have shown here that a similar inhibitory tone appears to control INs and PCs when no further distinction is made between the two PC sub-types, i.e. when IB and RS neurons are considered as a single population of principal (presumably excitatory) cells. However, among the three cell classes examined in this study, IBs are under the greatest tonic inhibition, whereas RSs and INs generate a comparable tonic current. These data suggest that while the activity of GABAergic and glutamatergic neurons may be overall balanced by a similar tonic control, differential modulation of PC sub-types provides fine-tuning of the excitatory drive, thus influencing the output modality of the subiculum. Cell type-specific expression of tonic inhibition is also seen in the thalamus [9], [21], in the cerebellum (Brickley et al., 1996) and in the neocortex, where experimental evidence indicates that this type of conductance is virtually absent in somatostatin-positive INs [12]. Interestingly, in the guinea pig CA1 hippocampal area tonic inhibition exerts a cell-type specific control that is somewhat complementary to what seen by us in the subiculum [7]. This may explain the greater excitability of subicular INs evidenced in our study.

Pharmacological blockade of GABA_A_R also resulted in a small but significant decrease of current σ^2^ in INs and RS cells, as opposed to IB neurons, which also exhibited uneven changes in current σ^2^. These data suggest that the biophysical properties of extra-synaptic GABA_A_R may differ between INs and PCs (see [19]), although the diverse electrotonic properties of these two cell classes may also justify our results. In light of this evidence, it would be therefore of interest to investigate in the near future if and to what extent sub-populations of GABA_A_R with different pharmacological profiles contribute to the inhibitory tone exerted over the three subicular cell classes.

### Phasic and tonic inhibition may play complementary roles in modulating subicular output modality

There is general consensus on the bursting nature of the subiculum, since systematic studies have consistently reported that IB cells are the most represented subicular PC. In this work, we report experimental evidence obtained from a similar number of IB and RS neurons (n = 18 and n = 17, respectively). The 1∶1 IB∶RS ratio characterizing our data set is likely due to the random choice of patched cells within the pyramidal cell layer of the subiculum (*cf.* Methods). Indeed, as previously evidenced by Menendez de La Prida [22], the different IB∶RS proportion is consequent to the sampling criteria.

Several studies have demonstrated that IB neurons are endowed with distinct intrinsic membrane properties that contribute to their bursting behaviour [5]. Consistent with this, we have shown here that subicular RS neurons could not burst in response to direct depolarization when GABA_A_R was pharmacologically blocked, whereas the bursting propensity of IB neurons was influenced. Further, it has been proposed that the two PC sub-types receive similar synaptic inputs [4], as also suggested by our finding of a comparable charge transfer. Thus, our results corroborate the view that intrinsic membrane properties play a major role in setting neuronal firing modality in response to excitatory and inhibitory inputs (*cf.*, [4]). It is intriguing to notice that picrotoxin treatment yielded opposite effects on the R_in_ of the two PC sub-types and it is also noteworthy that the effect of picrotoxin on R_in_ of IB cells is consistent with previous studies, regardless of [Cl^−^]_i_ (*cf.* [4], [9]). Indeed, it has been previously demonstrated that [Cl^−^]_i_ may still dynamically change in adult neurons even when experimentally set by whole-cell recording, due the expression of KCC2 [23]. Moreover, cell dialysis does not seem to affect the intrinsic bursting propensity of IB cells (*cf.* [24]). However, IPSPs evoked by local stimulation were able to break off action potential bursting of subicular PCs [4]. Therefore, in this context, tonic inhibition appears to be particularly relevant to the generation of bursting responses. We may expect that action potential burst is favoured by a relatively weaker inhibition in IB neurons as compared to non-bursting cells. Thus, the greater inhibitory tone exhibited by IB cells appears to be paradoxical. The role of tonic inhibition in IB neurons is indeed paradoxical in that it may favour the bursting behavior of these cells rather than decreasing their excitability. In support of our view, in the thalamus the inhibitory tone provided by GABA_A_R promotes low-threshold burst firing [9] and modulates burst-timing [21] of relay neurons. In line with this evidence, we have shown here that 9 out of 11 IB cells could no longer burst in response to direct depolarization following blockade of GABA_A_R. We may therefore hypothesize that whereas synaptic inhibition dampens the bursting propensity of subicular neurons through hyperpolarizing clamp, an inhibitory tone plays a paradoxically opposite role in promoting action potential burst. As the actual internal Cl^−^ concentration is unknown and cannot be completely controlled even in whole-cell recording (*cf.* [23]), we feel that, while our data should be extended with further studies, they still illustrate the action of picrotoxin at a set concentration, thus providing an initial evidence of the striking control of IB firing by tonic inhibition, similarly to what has already been shown for thalamic neurons [9].

### Network implications

It is well established that the subiculum gates hippocampal output activity and that GABA_A_R-mediated signalling contributes to this restraining function [3], [4]. In this context, Menendez de La Prida (2003) has proposed that local inhibition modulates subicular output activity by controlling the bursting propensity of IB cells [4]. Moreover, it has been reported that the subiculum provides an assorted output signal that is target-specific and depends on the local distribution of IB and RS neurons [25]. Witter (2006) has reported that subicular deep layers are mainly populated by IBs, whereas RS cells are mostly represented in the superficial layers [26]; moreover, the IB∶RS ratio increases along the proximal-distal axis of the subiculum [25]. This evidence suggests that both cell types contribute to subicular output along the “indirect” trisynaptic circuit, whereas RS neurons are the most represented PC within the “direct” temporo-ammonic route. This characteristic distribution of PC sub-types in the subiculum is relevant since it has been shown that rewiring along with impaired inhibition of subicular networks constitute a mechanism of temporal lobe epileptogenesis [16], [27]. Remarkably, we have recently found that tonic inhibition is paradoxically increased in rat subicular PCs immediately following pilocarpine-induced status epilepticus [28]. The involvement of tonic inhibition in epileptic syndromes is also evidenced in several animal models of absence epilepsy [29]. Moreover, enhancement of the inhibitory tone has also been described as a compensatory mechanism of increased neuronal excitability in Kv4.2 knock-out mice [30]. It would be therefore crucial to investigate whether cell type-specific changes in GABAergic currents occur in the subiculum following an epileptogenic insult.

### Concluding remarks

Our observations indicate that subicular INs are more excitable than PCs, as they are controlled by a smaller total synaptic inhibitory current and a weaker tonic inhibition. Remarkably, PCs receive sIPSCs at faster rate than INs. Moreover, the different expression of tonic inhibition within PC sub-types may play a central role in determining the bursting nature of subicular output activity. These observations corroborate the central role of GABAergic signalling in the gating function of the subiculum. We speculate that different sub-populations of INs may target functionally distinct subicular cell classes, which in turn would provide fine-tuned output responses by virtue of their intrinsic membrane properties. The apparent complementary roles of phasic and tonic inhibition may be relevant in the context of epileptic disorders.

## Supporting Information

Figure S1 (TIF)
