# Supplementary figures and images for: Cell Type-Specific Properties of Subicular GABAergic Currents Shape Hippocampal Output Firing Mode
Source: PLoS One. 2012 Dec 10;7(12):e50241. doi: 10.1371/journal.pone.0050241 (PMC3519474; doi:10.1371/journal.pone.0050241)

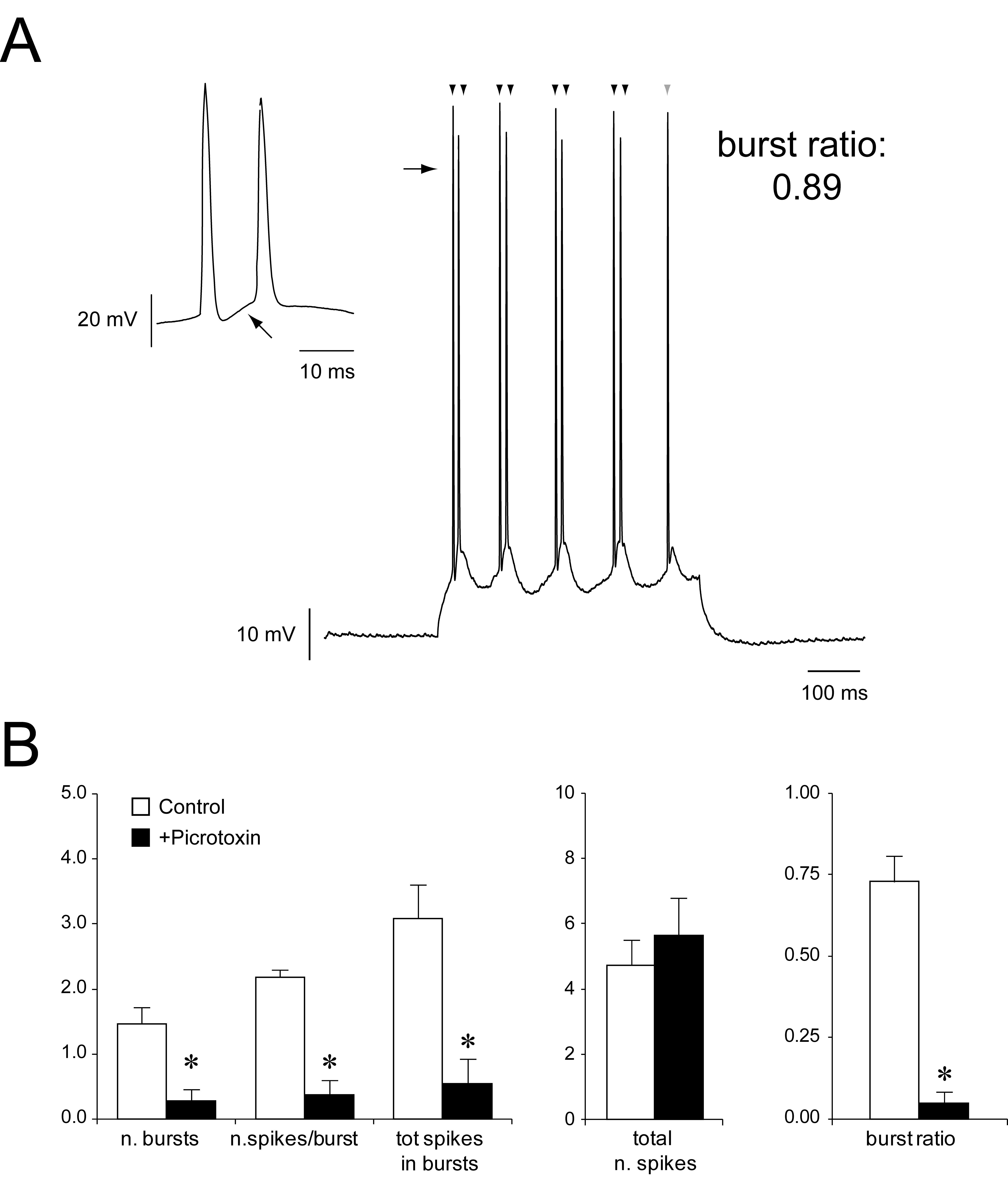

Supplement: Figure S1 — (TIF) [file pone.0050241.s001.tif]
